# The needle study: Machine learning as a new method for case‐finding in celiac disease

**DOI:** 10.1002/jpn3.70446

**Published:** 2026-04-27

**Authors:** Chiara Maria Trovato, Monica Montuori, Maria Ludovica Costanzo, Giusy Russo, Cosimo Ruggiero, Danila Volpe, Francesca Ferretti, Antonella Diamanti, Carlo Ciliberto, Salvatore Oliva

**Affiliations:** ^1^ Gastroenterology and Nutrition Unit Bambino Gesù Children Hospital, IRCCS Rome Italy; ^2^ Pediatric Gastroenterology and Liver Unit, Maternal and Child Health Department Sapienza University of Rome Rome Italy; ^3^ Department of Surgery “Sapienza” University of Rome Rome Italy; ^4^ UCL Centre for Artificial Intelligence University College London London UK

**Keywords:** artificial intelligence, children, diagnosis, uncommon feature

## Abstract

**Objectives:**

Despite a well‐defined diagnostic work‐up, uncertainties persist regarding celiac disease (CeD) detection strategies in the general population. Machine learning (ML) algorithms offer promise in aiding medical decision‐making on clinical data. This study aimed to utilize ML prediction models to identify uncommon features indicating the necessity for CeD screening in children.

**Methods:**

The discovery cohort comprised children with CeD exhibiting nonspecific clinical features, drawn from a single referral center, along with gender‐ and age‐matched controls. Data collected included demographic details, symptoms, laboratory tests, and family history of CeD or other autoimmune diseases, excluding anti‐transglutaminase immunoglobulin A (IgA) values. Various supervised ML models, utilizing input features to label CeD presence, were applied, with 73 features considered initially.

**Results:**

We collected data from 325 patients with CeD and 490 controls. A comprehensive evaluation using 10‐fold cross‐validation showed that a Ridge Classifier model achieved the best overall performance, with a mean area under the receiver operating characteristic curve of 0.763 (±0.070), F1‐score of 0.662 (±0.067), sensitivity of 0.652 (±0.110), and specificity of 0.689 (±0.081). The least absolute shrinkage and selection operator (LASSO) model identified a stable set of 40 predictive features, with noteworthy features including muscle pain, gastroesophageal reflux disease‐like symptoms, fatigue, and specific family histories of autoimmune disease.

**Conclusions:**

ML models, particularly the LASSO model, exhibited accuracy in identifying CeD in children with nonspecific clinical features. The identification of 40 uncommon features predictive of CeD diagnosis could support a more effective case‐finding strategy, potentially enhancing CeD detection.

## Abstract (highlights)

**What is Known:**

- Celiac disease (CeD) in children often presents with nonspecific or extraintestinal symptoms, contributing to underdiagnosis.
- Current diagnostic guidelines define how to confirm CeD but provide limited support for identifying which children should undergo serologic screening.
- Machine learning (ML) approaches in CeD have mainly focused on histology or imaging rather than clinical case‐finding.

**What is New:**

- A ML model using routine clinical data (excluding serology) identifies children at increased risk for CeD.
- Forty stable clinical, laboratory, and familial features associated with CeD were identified.
- This approach may support pediatricians in targeted case‐finding and earlier CeD detection.

## Graphical abstract

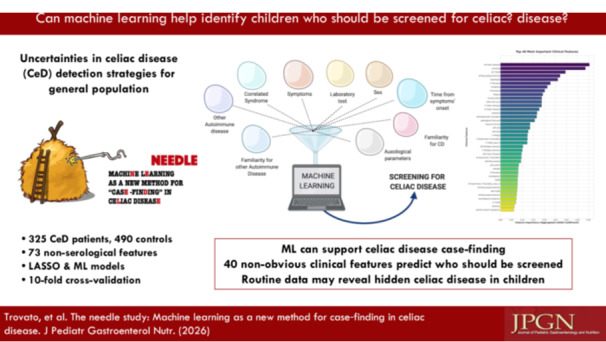

## 1 INTRODUCTION

Machine learning (ML) represents a convergence of statistics and computer science, offering the potential for robust support systems in medical decision‐making, particularly in diagnosing patients based on clinical data.

Common examples of ML in medicine include identifying thyroid or lung nodules in ultrasound or computed tomography (CT) images,^1^ or developing risk assessment models like the Framingham Risk Score for heart disease.^2^ ML has also helped developing and perfecting algorithms for several autoimmune diseases, including autoimmune thyroid disease and systemic lupus erythematosus.^3^ Celiac disease (CeD), as one of the most common autoimmune diseases, affecting more than 1% of the global population,^4^, ^5^ would greatly benefit from a ML‐enhanced screening algorithm. In fact, unsurprisingly, efforts to apply ML in CeD have increased, with several authors utilizing ML techniques to identify high risk patients,^6^, ^7^ recommend CeD screening,^8^ and determine predictive features for diagnosis.^9^ Additionally, a new ML model using automated image processing from capsule endoscopy has been proposed.^10^ Furthermore, a systematic review^11^ explored computer‐aided diagnosis of CeD through image processing techniques for detecting villous atrophy.

ML algorithms aim to standardize, simplify, and expedite decision processes following an evidence‐based approach, particularly benefiting physicians with limited experience and patients with challenging diagnosis.

CeD's clinical variability has earned it the moniker “the clinic chameleon,”^12^ often making prompt identification challenging. Given its varied clinical presentations, a definitive diagnosis is essential before prescribing a lifelong gluten‐free diet (GFD).^13^ Symptoms can encompass “typical” gastrointestinal issues like abdominal pain, constipation, diarrhea and bloating, as well as “atypical” systemic symptoms such as anemia, muscle pain, failure to thrive, migraine, and other neurological problems.^14^

Although diagnostic algorithms are well‐defined by several scientific societies,^15^, ^16^, ^17^ the debate over case‐finding versus mass screening for the general population remains open. As proposed by Popp in 2019,^18^ the most effective diagnostic improvement may come from screening known at‐risk groups or the entire population. While diagnosis is straightforward when autoantibodies are positive or symptoms are recognizable, identifying patients for screening remains a valuable tool for clinicians and the scientific community.

In this study, we aim to develop an ML model based on several parameters (uncommon symptoms, laboratory tests, familial history of CeD or other autoimmune diseases, gender, and auxological parameters), excluding antibody values, to subsequently identify the most relevant features that can be used to support clinicians in screening children despite the absence of a clear suspicion of CeD. The final goal is to develop and validate a probabilistic model that can be used to enhance the CeD diagnosis by applying a case‐finding strategy.

## 2 METHODS

### 2.1 Ethics statement

Data collection was in line with good clinical practice policies and the UE General Data Protection Regulation. The study protocol was defined in accordance with the Declaration of Helsinki and approved by the ethics committee of Policlinico Universitario Umberto I in Rome (ref: 1035/2021). All authors reviewed the study data and approved the final manuscript.

### 2.2 Study design

We constructed a discovery cohort comprising children with CeD presenting nonspecific clinical features. Samples were selected from a large cohort of children diagnosed with CeD at the Pediatric Gastroenterology and Liver Unit, Sapienza University of Rome, Italy, diagnosed during the period between 2014 and 2019. Controls were selected to be comparable to cases in terms of age and sex (frequency matching); however, no individual matching procedure was applied. For all patients, demographic and anthropometric characteristics, symptoms, laboratory tests, familial history of CeD or other autoimmune diseases were collected. Anti‐transglutaminase immunoglobulin A values (TGA‐IgA), suggestive of disease, were excluded. Symptoms were collected using a consistent, structured clinical interview routinely adopted at our center. Although not formally validated, the interview followed a predefined script covering gastrointestinal, systemic, neurological symptoms, and family history up to second‐degree relatives.

Details of the clinical data and laboratory tests considered in the ML model are reported in Table S1. Only patients aged 18 years or less and without a previous diagnosis of CeD were included in the study; additionally, only CeD patients with data available for all features were included. Patients with EoE and CeD, or with other infective diseases or with unknown family and/or medical history were excluded.

Data were processed through a supervised ML, serving as input features to predict the presence of CeD. The total number of input features amounts to 73. Data were standardized before being included in subsequent analysis.

Deviation from normality was assessed using the Shapiro–Wilk test of normality, as well as graphical distribution (quantile–quantile plot). Owing to the large sample size for this study, the sample was considered normally distributed in accordance with the Central Limit Theorem. Student's *t*‐test was used to compare continuous variables between the two independent groups, and the *χ*‐squared test was used to compare categorical variables between the two independent groups. All statistical analyses were performed in Python.

### 2.3 ML model development and evaluation

To ensure a robust evaluation of the models and to address concerns about data splitting, we implemented a comprehensive 10‐fold stratified cross‐validation strategy. The dataset was repeatedly split into 90% for training and 10% for testing, with the process repeated 10 times to ensure that every sample was in the test set exactly once. This approach provides a more reliable estimate of model performance on unseen data compared to a single train‐test split.

Given the large number of features available, we propose adopting the least absolute shrinkage and selection operator (LASSO)^19^ as the main ML model to predict the presence of CeD. The LASSO algorithm is designed to fit training data while simultaneously selecting the most relevant features to achieve accurate predictions. This is accomplished by solving a least‐squares minimization problem penalized with a L1‐norm regularization term (i.e., sum of the absolute values of the candidate linear estimator). Such regularization favors estimators with high sparsity (i.e., several zeros in the vector entries), thereby encouraging the activation of only the features most correlated with the output variable (in this case, the presence/absence of CeD).

More formally, let *x* denote a vector with *d* = 73 entries (or features) representing all the information collected for a single patient, and let *y* be a binary variable indicating whether the patient is CeD positive (*y* = 1 if CeD positive, *y* = –1 otherwise). Then, LASSO finds an estimator θ (namely a vector with the same number of entries as *x*) by minimizing the optimization problem

$$\text{mi}n_{\theta }\;\;\;1/n\;\;\sum_{i=1}^{n}{\left(\theta^{⊤}\;\cdot x_{i}-y_{i}\right)}^{2}+\lambda {\left|\left|\theta \right|\right|}_{1}$$

over the training set (comprising of *n* examples), with regularization parameter and ${\mathrm{||\cdot ||}}_{1}$ denoting the L1‐norm. The regularization term λ>0is chosen by model selection.

While the LASSO estimator aims to strike a balance between interpretability and performance, we also considered additional ML estimators: Support vector machines (SVM) with a linear kernel, ridge regression with L2 regularization, random forests, gradient boosting, and a decision tree. These estimators are introduced to evaluate and assess the overall accuracy achievable by an ML model on the present dataset.

Model performance was assessed using a suite of metrics to provide a holistic view beyond simple accuracy. These included the area under the receiver operating characteristic curve (AUC), F1‐score, precision (positive predictive value), and recall (sensitivity). The optimal classification threshold for each model was determined using Youden's *J* statistic on the cross‐validated ROC curve.

To validate the stability of the features selected by the LASSO model, we analyzed their selection consistency across all 10 folds of the cross‐validation (Figure S1). Feature consistency was defined as the frequency with which a feature was assigned a non‐zero coefficient during the training of each fold. A feature was considered stable if it was selected in at least 80% of the folds. Feature importance was quantified by calculating the mean absolute coefficient value for each feature across all 10 folds, providing a measure of the feature's impact on the model's predictions. All hyperparameters were tuned automatically within the cross‐validation loop. All analyses were performed in Python using the scikit‐learn library.^20^

### 2.4 Statistical analysis

We employed a bootstrapping approach to create 20 separate discovery and validation sets by randomly sampling our original dataset to maintain a one‐to‐one CeD: non‐CeD distribution. This sampling strategy mitigates biasing effects due to class imbalance. More precisely, we first sampled a test set consisting of approximately 20% of the total data, maintaining the same ratio of CeD and non‐CeD patients as the original dataset (*n* = 160: 40 CeD, 120 non‐CeD). Then, from the remaining 80% (640 records: 160 CeD, 480 non‐CeD), we created 20 discovery sets by randomly sampling with replacement from the discovery data. To achieve a 1:1 distribution of positive/negative samples in the discovery sets (420 CeD, 420 non‐CeD), CeD patients were oversampled and non‐CeD patients undersampled.

## 3 RESULTS

### 3.1 Study population

**Figure 1:**
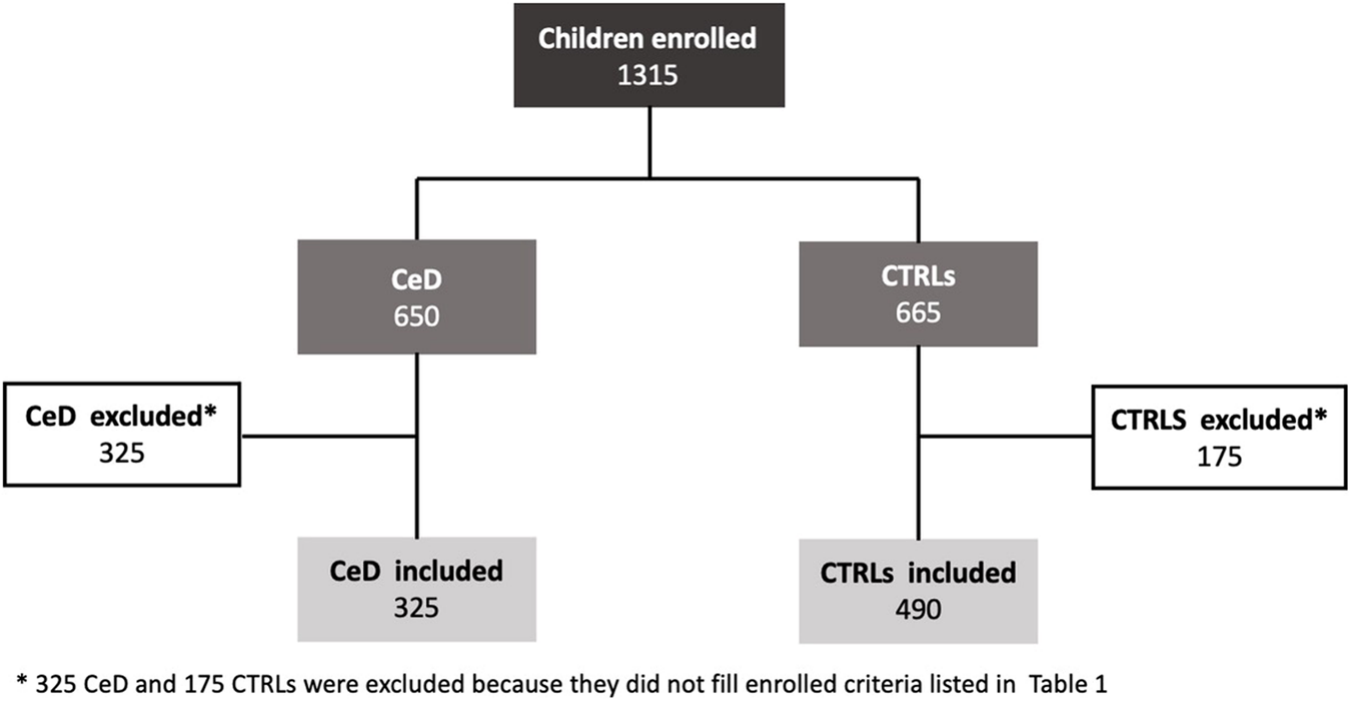
Flowchart illustrating patient selection, inclusion and exclusion criteria, and final composition of the study cohort. CeD, celiac disease; CTRLs, controls.

In the study cohort, initially, 1315 children were enrolled: 650 with CeD and 665 controls, matched by age and gender as specified above. Out of 650 children diagnosed with CeD between 2014 and 2019, 325 children with CeD (207 females, 118 males, with a mean age of 8.6 years) who met the inclusion criteria were enrolled in the study. The inclusion and exclusion flowchart is depicted in Figure 1. All cases of CeD were diagnosed according to the European Society for Pediatric Gastroenterology, Hepatology, and Nutrition (ESPGHAN) guideline.

The control group consisted of children hospitalized for other diseases at the Pediatric department of Policlinico Umberto I University Hospital in Rome between 2014 and 2019. Of the 665 initially selected, 175 had diarrhea, abdominal pain, and vomiting due to infectious diseases and were therefore excluded. Thus, 490 controls (239 females, 251 males, with a mean age of 10.4 years) were included (Table 1).

**Table 1:** Demographic, clinical, laboratory, and family history characteristics of the study cohort, including comorbid autoimmune conditions in first‐ and second‐degree relatives.

Variables	CeD group	Controls	*p*
	325	490	
Gender			
Female	207 (63.7%)	239 (48.8%)	**<0.001**
Male	118 (36.3%)	251 (51.2%)	
Age			
Mean age	8.67	10.47	**<0.001**
Median age	9.00	11.00	
Auxological data			
Height (mean)	130 cm	133 cm	0.1
Weight (mean)	31.7 kg	36.9 kg	**<0.001**
Symptoms			
Recurrent abdominal pain	157 (48%)	186 (38%)	**0.004**
Diarrhea	68 (20.9%)	118 (24%)	0.293
Constipation	96 (29.5%)	104 (21.2%)	**0.007**
Dysfagia	2 (0.6%)	6 (1.2%)	**<0.001**
Apthosis	2 (0.6%)	4 (0.8%)	**0.004**
Vomiting	26 (8%)	112 (22.8%)	**<0.001**
Reflux‐like symptoms	64 (19.6%)	171 (34.8%)	**<0.001**
Failure to thrive	89 (27.3%)	70 (14%)	**<0.001**
Joint pains	39 (12%)	39 (7.9%)	0.053
Muscle pains	24 (7.3%)	24 (4.8%)	0.140
Asthenia	41 (12.6%)	44 (8.9%)	0.096
Fatigue	2 (0.6%)	6 (1.2%)	0.388
Alopecia	3 (0.9%)	0	**0.033**
Dental caries	0	3 (0.61%)	0.158
Dental enamel hypoplasia	2 (0.6%)	1 (0.2%)	0.342
Migraine	49 (15%)	55 (11%)	0.107
Irritability	9 (2.7%)	15 (3%)	0.809
Sleep disorder	22 (6.7%)	54 (11%)	**0.041**
Neurological symptoms	20 (6.1%)	78 (15.9%)	**<0.01**
Osteopenia	6 (1.8%)	14 (2.8%)	0.312
Osteoporosis	2 (0.6%)	4 (0.8%)	0.745
Pathological fractures	1 (0.3%)	6 (1.2%)	0.165
Time from symptom's onset (months, mean)	27.9 m	27.2 m	0.794
Associated autoimmune disease			
Type 1 diabetes	7 (2.1%)	5 (1%)	0.193
Hashimoto's thyroiditis	11 (3.3%)	9 (1.8%)	0.168
Rheumatoid arthritis	1	0	0.222
Systemic lupus erythematosus	0	0	
Psoriasis	0	3 (0.6%)	0.156
Vitiligo	0	0	
Multiple sclerosis	0	0	
Related syndrome			
Down syndrome	1 (0.3%)	2 (0.4%)	0.811
William's syndrome	0	0	
Turner syndrome	0	1 (0.2%)	0.419
Familiarity for other autoimmune disease			
− *Mother:*			
CeD	40 (12.6%)	7 (1.53%)	**<0.01**
Type 1 diabetes	1 (0.3%)	1 (0.2%)	0.819
Hashimoto's thyroiditis	30 (9.2%)	35 (7.8%)	0.479
Rheumatoid arthritis	2(0.6%)	2(0.4%)	0.476
Systemic lupus erythematosus	0	1 (0.2%)	0.394
Psoriasis	2 (0.6%)	5(1.1%)	0.471
Vitiligo	1 (0.3%)	1 (0.2%)	0.820
Multiple sclerosis	1 (0.3%)	1 (0.2%)	0.819
− *Father*			
CeD	15 (4.8%)	5 (1.16%)	**0.002**
Type 1 diabetes	5 (1.53%)	5 (1.19%)	0.438
Hashimoto's thyroiditis	3 (0.9%)	5 (1.19%)	0.798
Rheumatoid arthritis	1 (0.3%)	4 (0.8%)	0.317
Systemic lupus erythematosus	0	1 (0.2%)	0.349
Psoriasis	4 (1.23%)	10 (2.23%)	0.303
Vitiligo	1 (0.3%)	5 (1.21%)	0.206
Multiple sclerosis	0	3 (0.67%)	0.140
− *Sister*			
CeD	17 (5.5%)	6 (1.3%)	**0.001**
Type 1 diabetes	2 (0.6%)	1 (0.2%)	0.386
Hashimoto's thyroiditis	1 (0.3%)	3 (0.6%)	0.489
Rheumatoid arthritis	0	1 (0.2%)	0.394
Systemic lupus erythematosus	0	1 (0.2%)	0.395
Psoriasis	0	3 (0.6%)	0.140
Vitiligo	0	1 (0.2%)	0.485
Multiple sclerosis	0	1 (0.2%)	0.394
− *Brother*			
CeD	22 (6.8%)	3 (0.6%)	**<0.001**
Type 1 diabetes	0	1 (0.2%)	0.088
Hashimoto's thyroiditis	2 (0.6%)	3 (0.6%)	0.927
Rheumatoid arthritis	0	3 (0.6%)	0.140
Systemic lupus erythematosus	0	1 (0.2%)	0.394
Psoriasis	1 (0.3%)	1 (0.2%)	0.819
Vitiligo	0	2 (0.4%)	0.228
Multiple sclerosis	0	1 (0.2%)	0.394
Laboratory tests (mean)			
Hemoglobin (g/dL)	12.6	13.1	0.117
White cell count (10^3^/μL)	7.391	8.484	0.238
Absolute number of lymphocytes (10^3^/μL)	3.061	3.016	0.833
Absolute number of neutrophilis (10^3^/μL)	3.602	4.674	0.067
Platelets (10^3^/μL)	296.8	296.7	0.989
Mean cell volume (fl)	79.6	81.6	**<0.01**
Red blood cell distribution width (%)	14.5	13.3	0.234
Ferritin (ng/mL)	35.3	62.8	0.222
Lactate dehydrogenase (U/L)	241	227	0.060
Creatine phosphokinase (U/L)	123	133	0.429
Protein (g/dL)	7.1	7.2	0.437
Albumin (g/dL)	4.7	4.7	0.150
Triglyceride (md/dL)	1.06	0.83	0.259
Total IgA (g/L)	1.2	2.11	0.165
IgM (g/L)	1.1	1.2	0.287
IgG (g/L)	9.8	10.6	**0.006**
Prothrombin time (%)	12	17	**<0.01**
Partial thromboplastin time (s)	32	31	0.547
INR (ratio)	1.1	1.07	0.267

*Note*: Bold values indicate statistically significant.

Abbreviations: CeD, celiac disease; IgA, immunoglobulin A; IgG, immunoglobulin G; IgM, immunoglobulin M; INR, international normalized ratio.

The most frequently reported pathologies in the control group were eosinophilic esophagitis (EoE); inflammatory bowel diseases (IBD), pneumonia, epilepsy, anal fissures, gastroesophageal reflux disease (GERD); eating behavior disorders, irritable bowel syndrome, hypertransaminasemia, hypothyroidism, urticaria, and food allergy.

### 3.2 Demographic and clinical data

Table 1 presents the clinical data for both populations. Significant disparities in gender, age, and weight were evident between the two groups. In terms of signs and symptoms, a noteworthy discrepancy was noted in the prevalence of recurrent abdominal pain, which was markedly higher in the CeD group. Additionally, variations were observed in the occurrence of constipation, dysphagia, aphthous ulcers, vomiting, GERD‐like symptoms, poor growth, alopecia, sleep disorders, and neurological symptoms. In terms of family history of autoimmune diseases, patients with CeD exhibited a notably higher frequency of first‐degree relatives with CeD, Hashimoto's thyroiditis, psoriasis, and CeD in other family members. Furthermore, concerning laboratory values, statistically significant differences were observed in the mean corpuscular volume (MCV), prothrombin time (PT), and total IgG levels between the two groups.

### 3.3 Performance of the ML models

**Figure 2:**
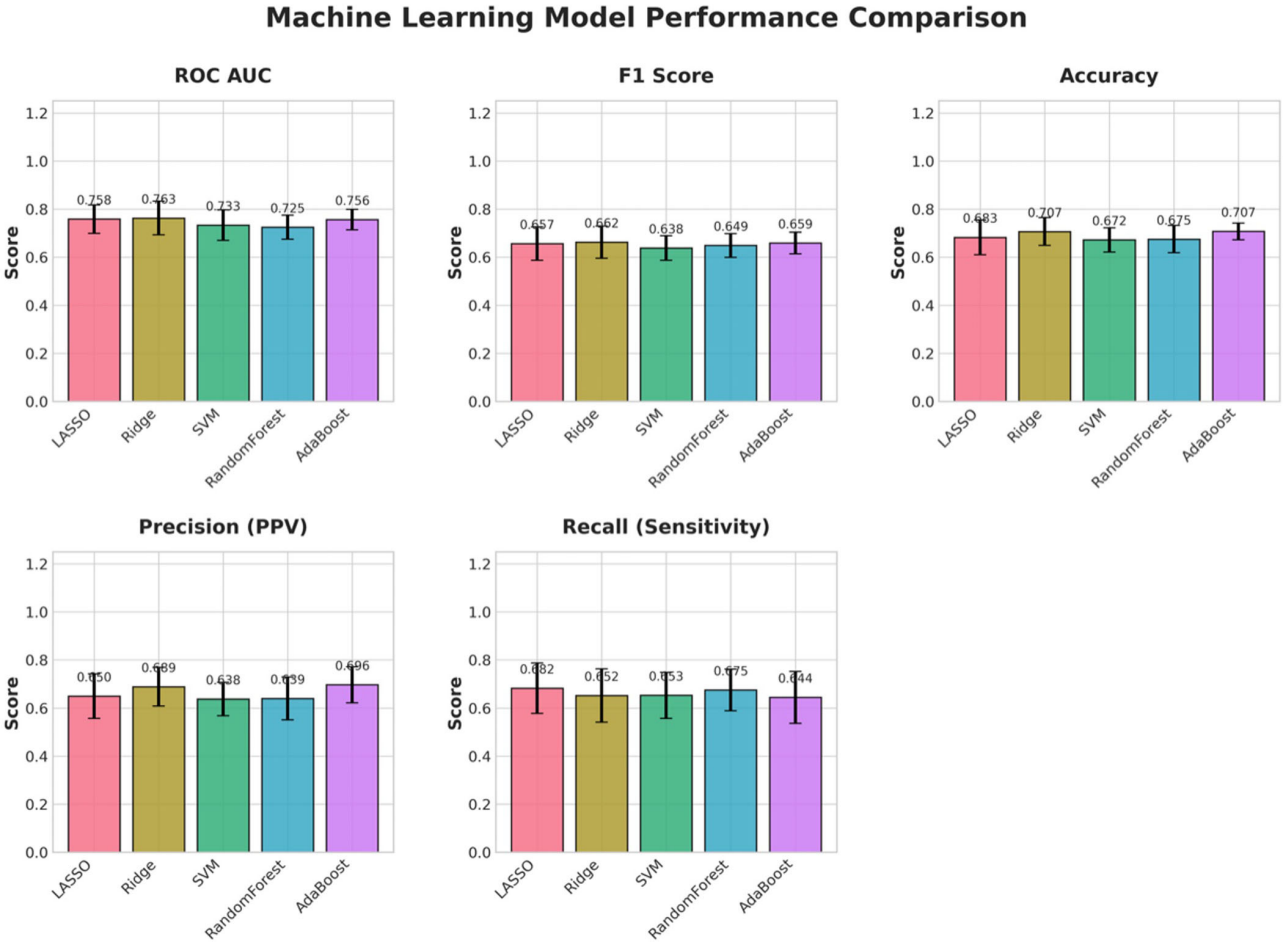
Comparative performance of all machine‐learning models evaluated using 10‐fold cross‐validation, across five key performance metrics. LASSO, least absolute shrinkage and selection operator; ROC AUC, area under the receiver operating characteristic curve; SVM, support vector machines.

**Figure 3:**
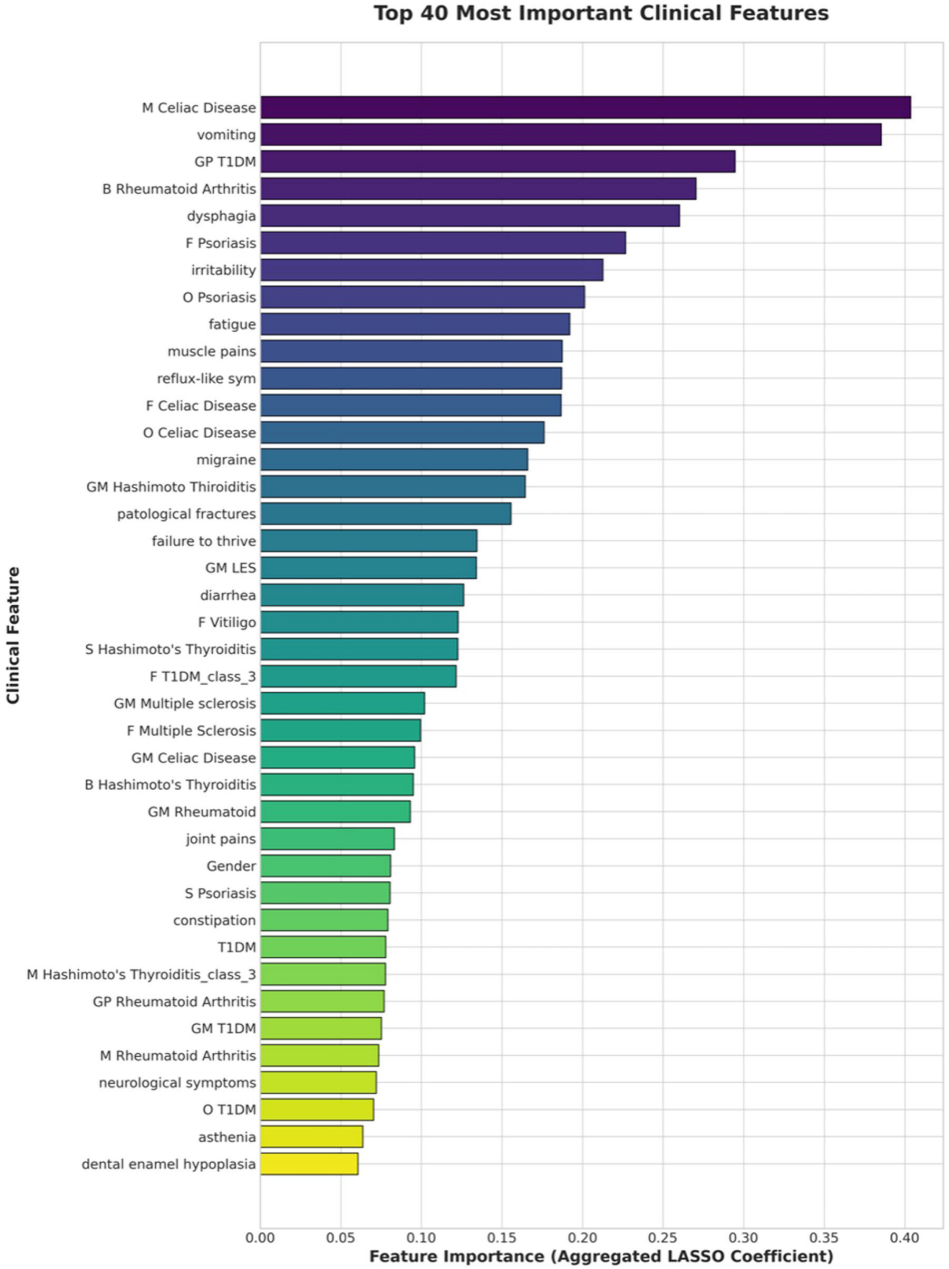
Top 40 most important clinical features identified by the LASSO model, ranked according to their aggregated importance score across cross‐validation folds. B, brother; F, father; GM, grandmother; GP, grandparent; LASSO, least absolute shrinkage and selection operator; LES, lower esophageal sphincter; M, mother; O, other; S, sister; T1DM, type 1 diabetes mellitus.

The performance of all models was assessed using 10‐fold cross‐validation, with results summarized in Figure 2. While several models showed competitive performance, the Ridge classifier emerged as the most balanced, achieving the highest mean F1‐score (0.662) and a mean AUC of 0.763. The LASSO model, while slightly lower in predictive metrics, provided crucial insights through its feature selection capabilities (Figure S1). Across the cross‐validation folds, LASSO identified a highly stable set of features predictive of a CeD diagnosis. The 40 most important of these features are shown in Figure 3, with their selection consistency detailed in Figure S2. Key predictive features included a family history of autoimmune diseases (Hashimoto's, Psoriasis), specific symptoms (muscle pain, fatigue, GERD‐like symptoms), and certain lab value deviations (Figures 3 and S3).

## 4 DISCUSSION

This study holds particular significance as it focuses on the identification of uncommon features of CeD using ML models to enable early diagnosis of this condition. Recognizing signs and symptoms suggestive of CeD could prove invaluable in identifying undiagnosed celiac patients. Our ML algorithm demonstrated that the LASSO model was the most accurate in identifying 40 uncommon features predictive of a CeD diagnosis. These findings could support a case‐finding strategy in clinical practice.

To date, screening for CeD in the general population remains a significant challenge. The ongoing debate between mass screening versus case‐finding continues, with recent data from Italy shedding light on the role of mass screening in primary schools.^5^ However, on the other hand, it is widely known that screening should be considered mostly for selective populations.

In this regard, our results reveal different parameters that can predict the presence of CeD in routine clinical practice by identifying patients who might have a higher risk of testing positive to the CeD serology. Our model indicates that symptoms like sleep disorders, muscle pain, and fatigue can be predictive of a CeD diagnosis. Among laboratory tests, our model highlights the significant role of low levels of triglycerides, an increase in RDW, and an absolute number of eosinophils. Regarding family history, our algorithm outlines the role of autoimmune diseases not only in first‐degree relatives (parents and siblings), but also in second‐degree relatives such as grandparents and uncles. The majority of autoimmune diseases represented are Hashimoto Thyroiditis and Rheumatoid Arthritis.

In this model, we have intentionally excluded anti‐transglutaminase values due to their well‐known association with intestinal atrophy and CeD, observed in patients with elevated as well as low titers.^21^ The rationale of such a choice is to guide general pediatricians, who evaluate children commonly for non‐specific symptoms, to early identify predictors of CeD that can justify the screening. Therefore, our model includes routine laboratory tests, usually measured for other reasons but often altered in CeD (such as increased lactate dehydrogenase or creatine phosphokinase levels, or reduced serum ferritin or hemoglobin), which can serve as useful tools in predicting CeD diagnosis by prompting individuals for antibody screening. We believe that this prediction model can improve clinical approaches by identifying new “red flags” for CeD and suggesting screening in the general population. The proposed approach would allow diagnosis based on case‐finding strategy, therefore reducing the need for mass screen campaigns. The major aim of the scientific community is to diagnose as many patients as possible by discovering the hidden part of the “CeD iceberg” and diminishing the “submerged” undiagnosed and untreated patients. On the other hand, any screening strategy should be sustainable by reducing the costs and increasing the probability of early detection of the disease.

In literature some studies^22^ demonstrate how the application of ML models can support the diagnosis of CeD, but these studies mainly concern the histological diagnosis of CeD^23^, ^24^, ^25^, ^26^ and the use of the video capsule^10^, ^27^, ^28^ as a diagnostic tool. Based on our knowledge, this is the first study that uses ML to support clinical information for identifying the patient who should be screened for CeD.

To address the concern of excluding patients with incomplete data, we conducted a sensitivity analysis comparing our complete‐case approach to several data imputation strategies (mean, median, and most frequent imputation), with the results shown in Figure S4. The analysis showed no significant improvement in model performance with imputation, supporting the validity of the complete‐case analysis for this dataset. Furthermore, a benchmark analysis including TGA‐IgA values was performed (Figure S5). As expected, a model including TGA‐IgA showed higher predictive power (AUC 0.85), confirming the biomarker's importance. However, our clinical feature‐based model still demonstrated significant predictive value (AUC 0.76), highlighting its utility in scenarios where TGA‐IgA is not available or has not yet been ordered.

This study has several limitations. First, the main limitation is related to the potential pitfall of all ML projects, which lies in the uncertainty of the “exportability” of the model. Clinical data are derived from patients’ consultations; hence, awareness of the signs and symptoms depends on the clinical expertise of the clinician recording the data in the patients’ charts. On the other hand, in our discovery cohort, we selected only those patients who had non‐specific symptoms, while we excluded patients who had a clinical history easily suggestive of CeD. This cohort of patients represents a challenge even for expert physicians in the field. Another limitation lies in the different types of commercial kits available for laboratory tests. For this reason, normalization based on the upper limit of normal can be a useful strategy. However, the majority of the laboratory tests considered in our model were routine laboratory tests that usually do not exhibit significant variability in the normal ranges. Another possible pitfall is that our model might be applicable only within a selected age range, specifically childhood, and in a selected geographical region. Consequently, even if validated, this prediction score might be uniquely applicable in the pediatric population in our country, and future studies would be necessary to confirm its reliability. While bootstrapping can increase validity, the study could have benefitted by an external validation (e.g., involving data from a different center), or by including a different percentage of patients in the test set. In summary, this study developed a ML model based on a large number of clinical parameters (such as uncommon symptoms, data from family history, and laboratory tests) to facilitate early detection of CeD in children.

## 5 CONCLUSION

In children presenting with nonspecific clinical features, a ML‐based approach using routine clinical, laboratory, and familial data, independent of celiac serology, may effectively identify individuals at increased risk for CeD. The identification of stable and uncommon predictive features supports a targeted case‐finding strategy, potentially facilitating earlier diagnosis and reducing the burden of undetected disease. Further external validation is warranted to confirm the generalizability of this model across different pediatric populations and clinical settings.

## CONFLICT OF INTEREST STATEMENT

The authors declare no conflicts of interest.

## Supporting information

Supplemental Figure 1

Supplemental Figure 2

Supplemental Figure 3

Supplemental Figure 4

Supplemental Figure 5

Supplemental Table 1
