# Supplementary figures and images for: The needle study: Machine learning as a new method for case‐finding in celiac disease
Source: J Pediatr Gastroenterol Nutr. 2026 Apr 27;83(1):135–44. doi: 10.1002/jpn3.70446 (PMC13342765; doi:10.1002/jpn3.70446)

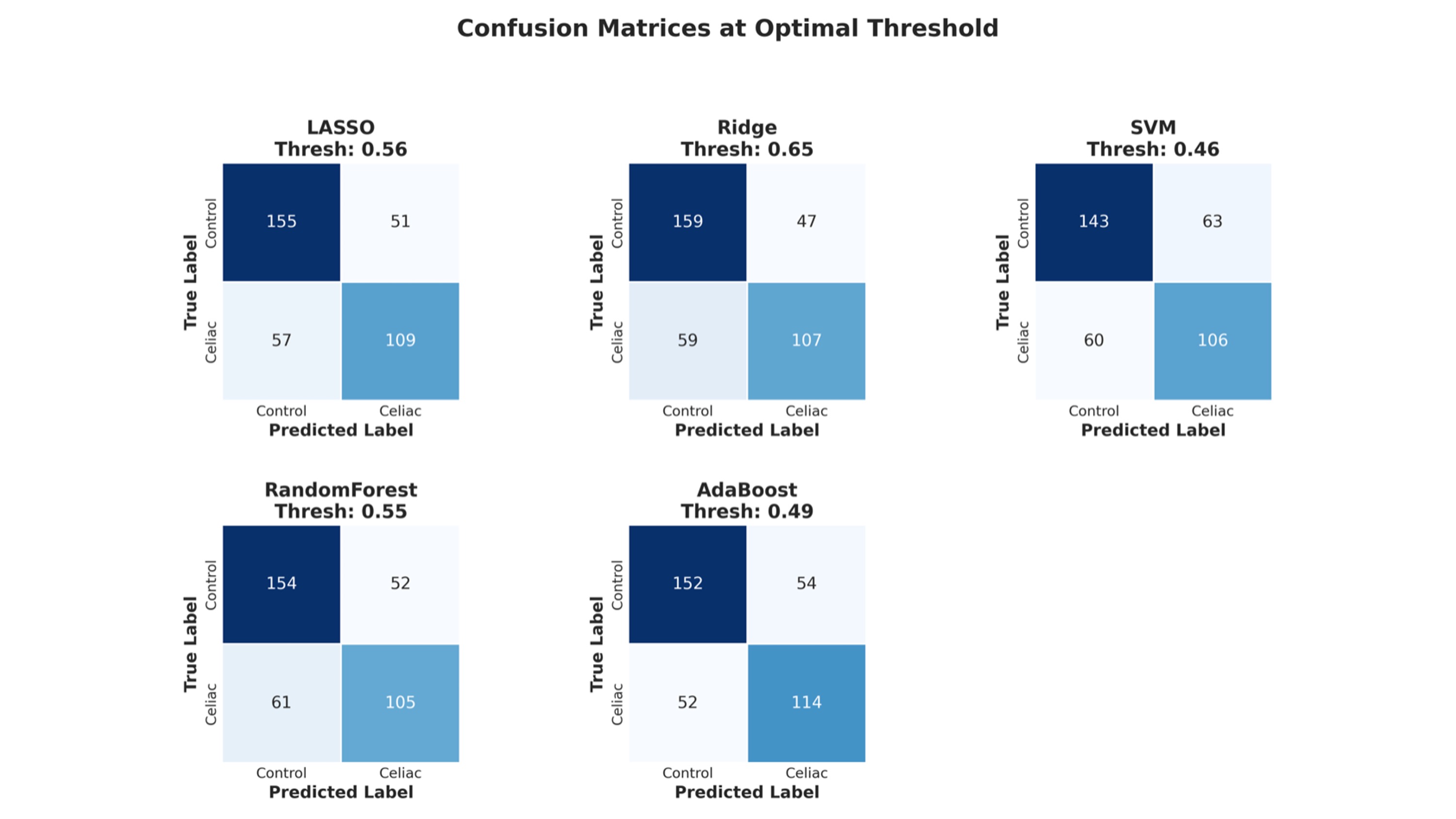

Supplement: Supplementary file 1 — Supplemental Figure 1 [file JPN3-83-135-s002.jpg]

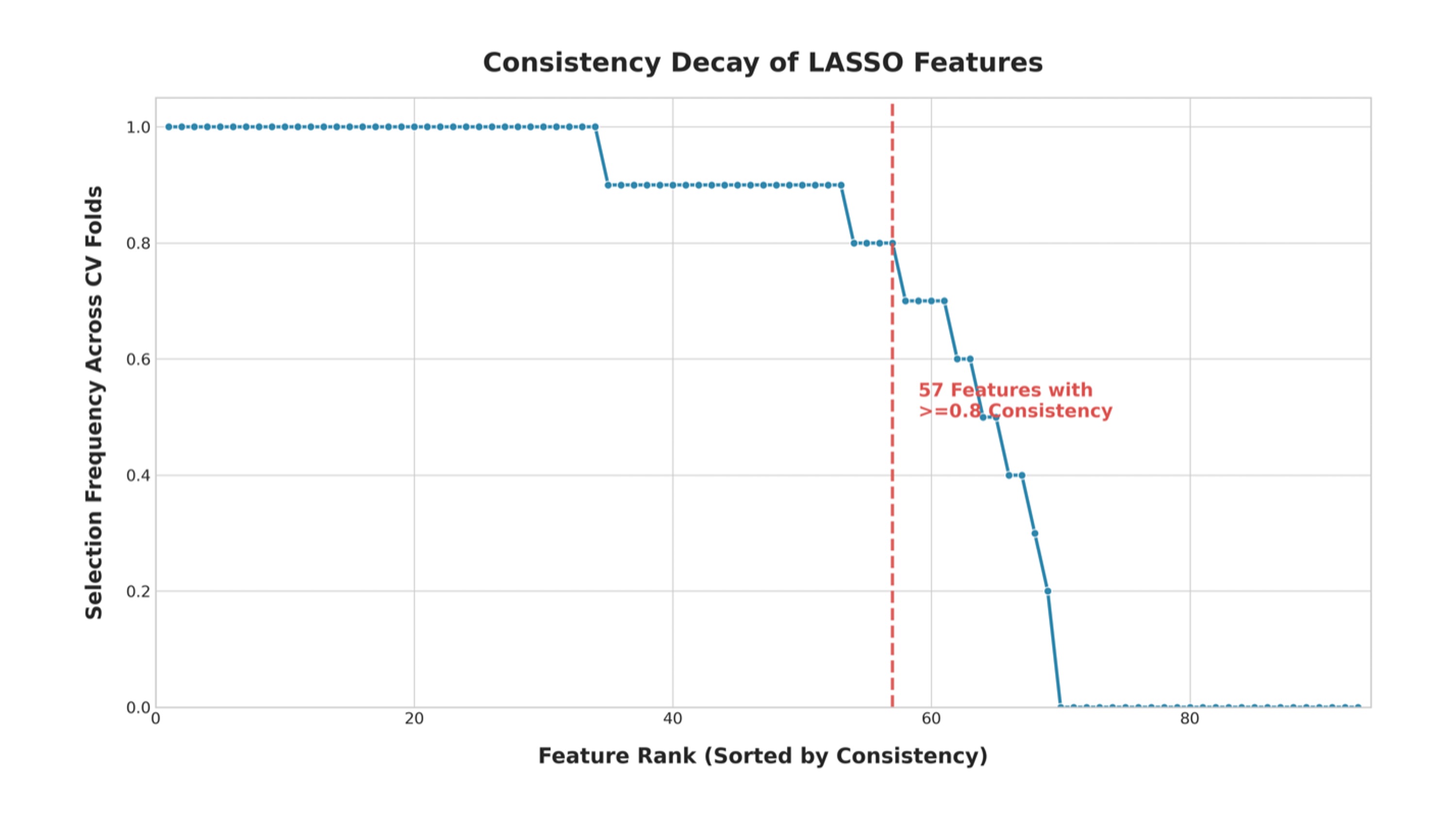

Supplement: Supplementary file 2 — Supplemental Figure 2 [file JPN3-83-135-s004.jpg]

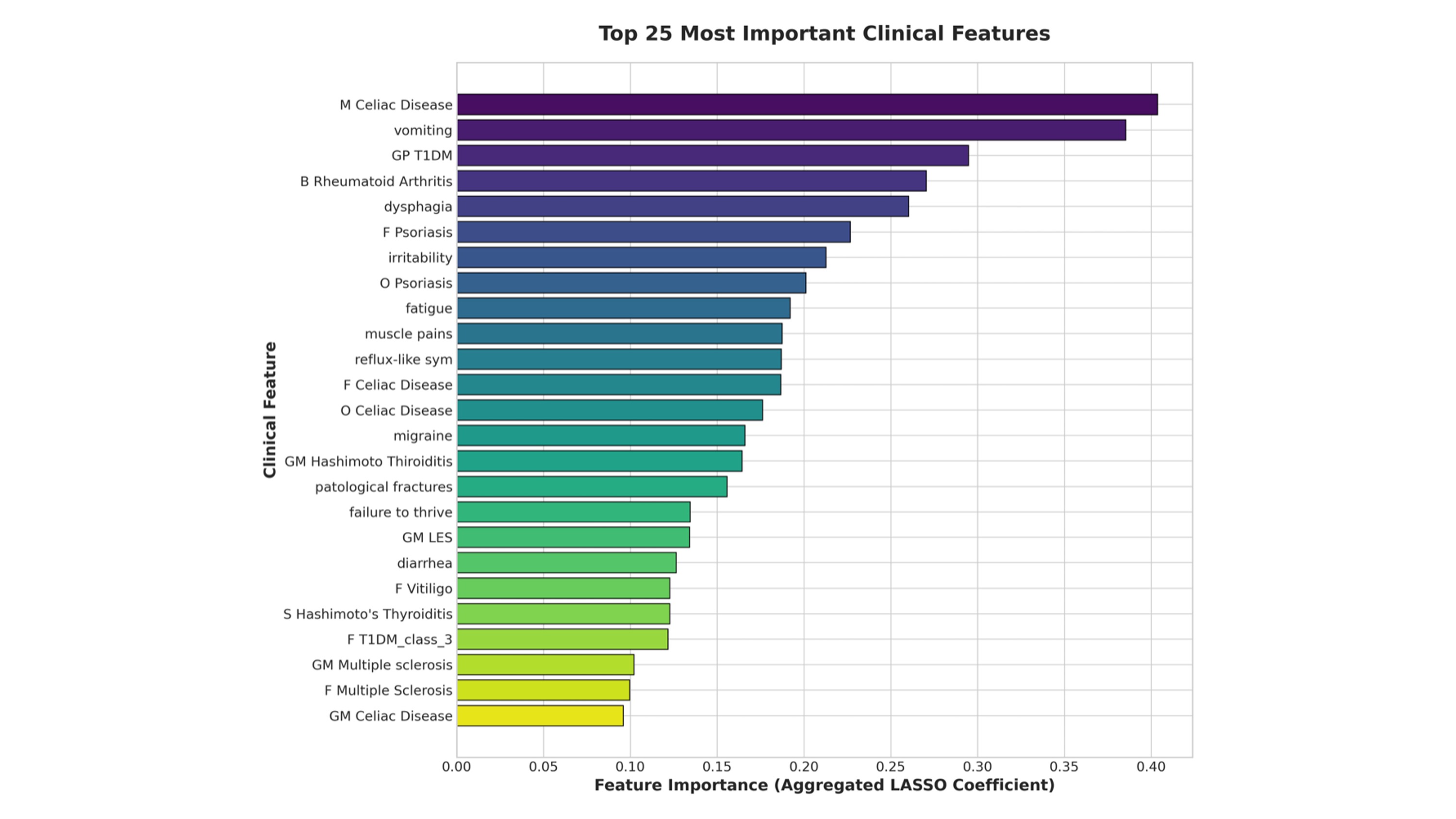

Supplement: Supplementary file 3 — Supplemental Figure 3 [file JPN3-83-135-s001.jpg]

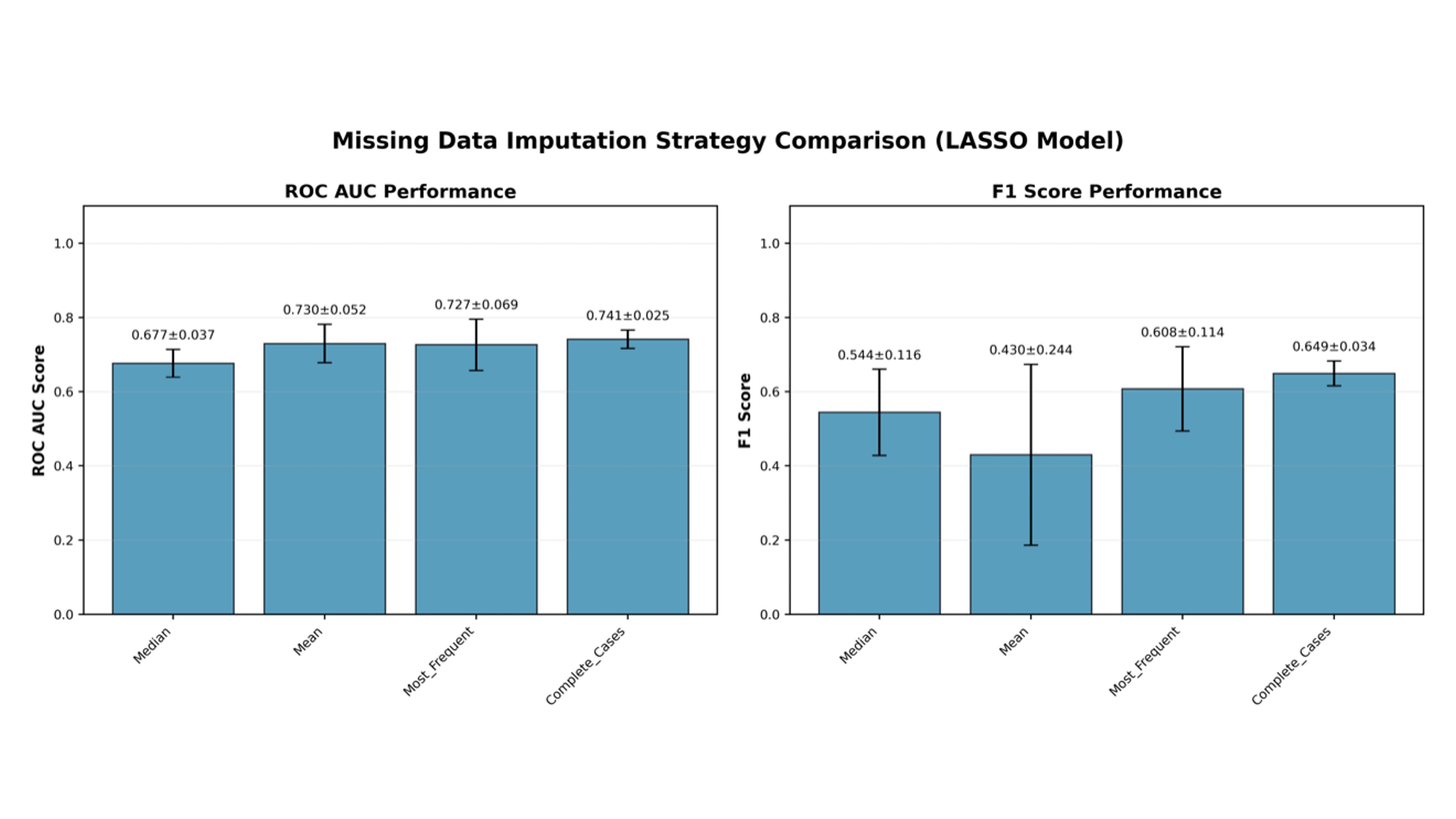

Supplement: Supplementary file 4 — Supplemental Figure 4 [file JPN3-83-135-s005.jpg]

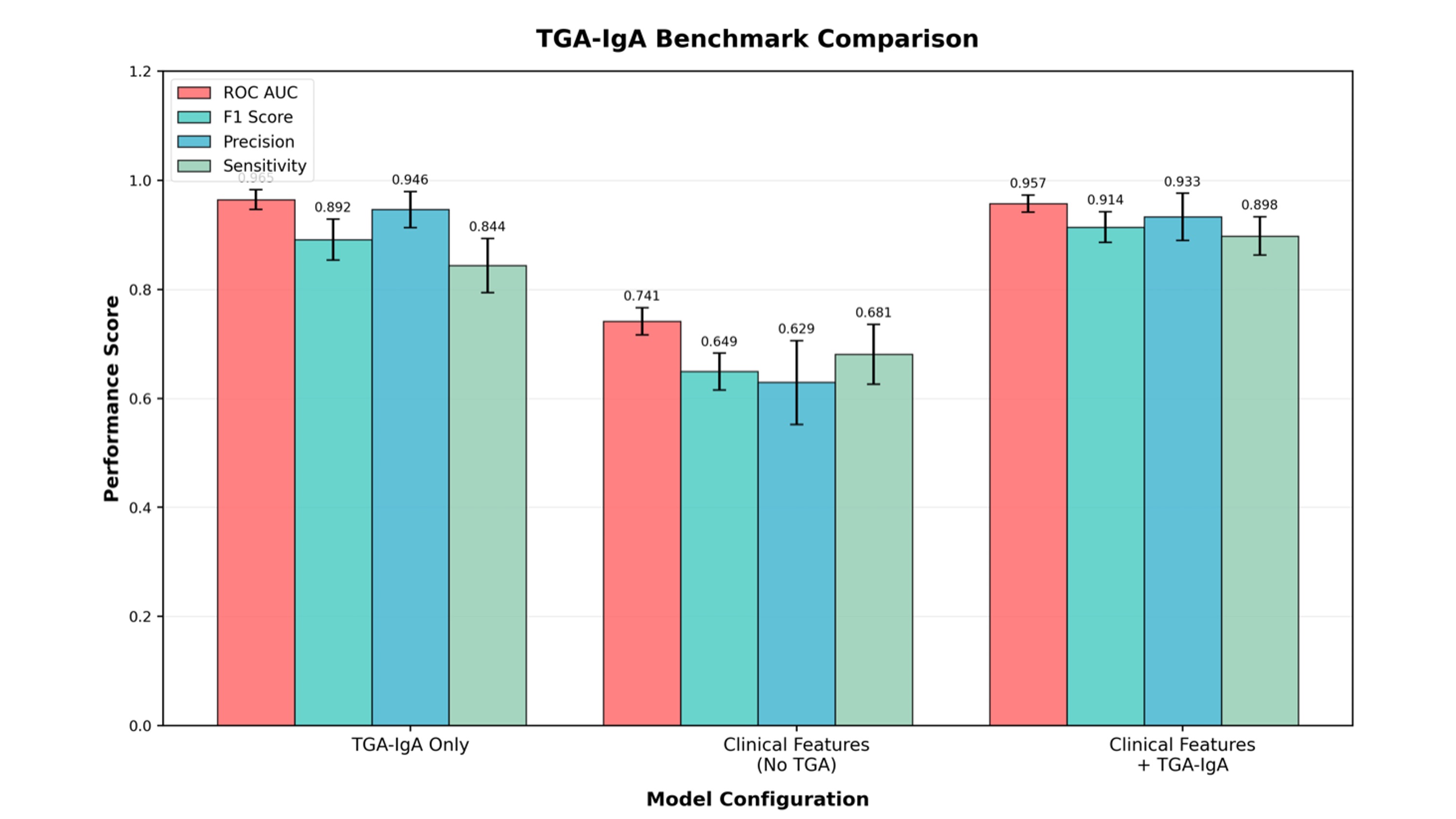

Supplement: Supplementary file 5 — Supplemental Figure 5 [file JPN3-83-135-s006.jpg]
