# Supplementary material for: The needle study: Machine learning as a new method for case‐finding in celiac disease
Source: J Pediatr Gastroenterol Nutr. 2026 Apr 27;83(1):135–44. doi: 10.1002/jpn3.70446 (PMC13342765; doi:10.1002/jpn3.70446)
Supplement: Supplementary file 6 — Supplemental Table 1 [file JPN3-83-135-s003.docx]

**SUPPLEMENTARY TABLE 1:** Detail of clinical data considered for enrollment.

| CATEGORY | VARIABLES |
| --- | --- |
| GENDER | Male/Female |
| AUXOLOGICAL DATA | Weight |
|  | Length/height |
|  | centiles |
| SYMPTOMS | Recurrent Abdominal Pain |
|  | Bloating |
|  | Diarrhea |
|  | Constipation |
|  | Aphthous |
|  | Vomiting |
|  | Reflux-like symptoms |
|  | Failure to thrive |
|  | Joints pain |
|  | Muscle pain (particularly lower limbs) |
|  | Asthenia |
|  | Fatigued |
|  | Alopecia |
|  | Dental caries |
|  | Dental enamel hypoplasia |
|  | Migraine |
|  | Irritability |
|  | Sleep disorder |
|  | Neurological symptoms |
|  | Osteopenia |
|  | Osteoporosis |
|  | Pathological Fractures |
| AUTOIMMUNE DISEASES | Type 1 Diabetes |
|  | Hashimoto’s thyroiditis |
|  | Rheumatoid arthritis |
|  | Systemic lupus erythematosus |
|  | Psoriasis |
|  | Vitiligo |
|  | Multiple Sclerosis |
| RELATED SYNDROMES | Down Syndrome |
|  | William Syndrome |
|  | Turner Syndrome |
| TIME FROM SYMPTOMS ONSET | Months |
| FAMILIARITY FOR CELIAC DISEASE | Family member affected (mother, father, sister, brother, grandparents, cousins, uncles) |
|  | Number of family member affected (1, 2, 3…) |
| FAMILIARITY FOR AUTOIMMUNE DISEASES | Type 1 Diabetes |
|  | Hashimoto’s thyroiditis |
|  | Rheumatoid arthritis |
|  | Systemic lupus erythematosus |
|  | Psoriasis |
|  | Vitiligo |
|  | Multiple Sclerosis |
| LABORATORY TEST | *Hematology*: Hemoglobin [g/dL]; White cell count, Lymphocytes, Neutrophilis, Platelets [10^3/microL]; Mean cell volume [fl]; Red Blood Cell Distribution Width [%] |
|  | *Coagulation*: Prothrombin time [%]; INR [ratio]; Partial thromboplastin time [sec] |
|  | *Chemistry*: Ferritin [ng/mL]; Lactate Dehydrogenase [U/L]; Creatine Phosphokinase [U/L]; Protein [g/dL]; Albumin [g/dL]; Triglyceride [md/dL]; Total IgA [g/L]; IgM [g/L]; IgG [g/L] |
